# Supplementary material for: Adaptation optimizes sensory encoding for future stimuli
Source: PLoS Comput Biol. 2025 Jan 17;21(1):e1012746. doi: 10.1371/journal.pcbi.1012746 (PMC11771873; doi:10.1371/journal.pcbi.1012746)
Supplement: S1 Text — Individual subjects’ data and fitting parameters; detailed natural scene statistics. (PDF) [file pcbi.1012746.s001.pdf]

Supplementary information

Parameter	Subj 1	Subj 2	Subj 3	Subj 4	Subj 5
**Control**					
κ_i: sensory noise	191.04	128.63	25.44	60.54	128.46
k: prior intensity	0.17	0.24	0.12	0.25	0.15
κ: prior width	14.73	22.47	15.49	14.69	16.83
**2-peak**					
k_1: kernel strength	0.05	0.08	0.09	0.11	0.08
κ_1: kernel width	74.8	77.2	39.7	31.8	105.7
k_2: kernel strength	0.13	0.11	49.47	0.25	49.49
κ_2: kernel width	3.17	2.32	0.0046	2.17	0.0071

Supplementary Table A: Best-fitting model parameters for every subject.

Parameter	Subj 1	Subj 2	Subj 3	Subj 4	Subj 5
**1-peak**					
k_1: kernel strength	0.04	0.07	0.07	0.07	0.06
κ_1: kernel width	127.2	93.4	64.2	77.0	182.8
**2-peak + Fisher**					
κ_i^a: sensory noise (adapt)	185.08	143.58	25.43	48.33	108.98
k_1: kernel strength	0.05	0.08	0.09	0.12	0.08
κ_1: kernel width	74.4	78.4	39.7	30.4	108.9
k_2: kernel strength	0.12	0.17	47.23	0.18	1.15
κ_2: kernel width	3.53	1.52	0.0048	2.45	0.27
**2-peak + kernel**					
k_1^{45}: 45° kernel strength	0.05	0.09	0.07	0.09	0.08
κ_1^{45}: 45° kernel width	134.6	75.2	92.0	49.2	92.3
k_2^{45}: 45° kernel strength	0.13	0.01	0.09	0.21	0.39
κ_2^{45}: 45° kernel width	5.21	700.0	7.53	7.52	1.22
$k_1^{22.5}$: 22.5° kernel strength	0.06	0.07	0.12	0.09	0.07
$\kappa_1^{22.5}$: 22.5° kernel width	35.8	79.1	22.9	46.4	191.9
$k_2^{22.5}$: 22.5° kernel strength	0.12	0.19	415	0.35	381
$\kappa_2^{22.5}$: 22.5° kernel width	1.96	1.27	5.7e-4	0.71	7.6e-4

Supplementary Table B: Best-fitting model parameters for every subject - alternative models.

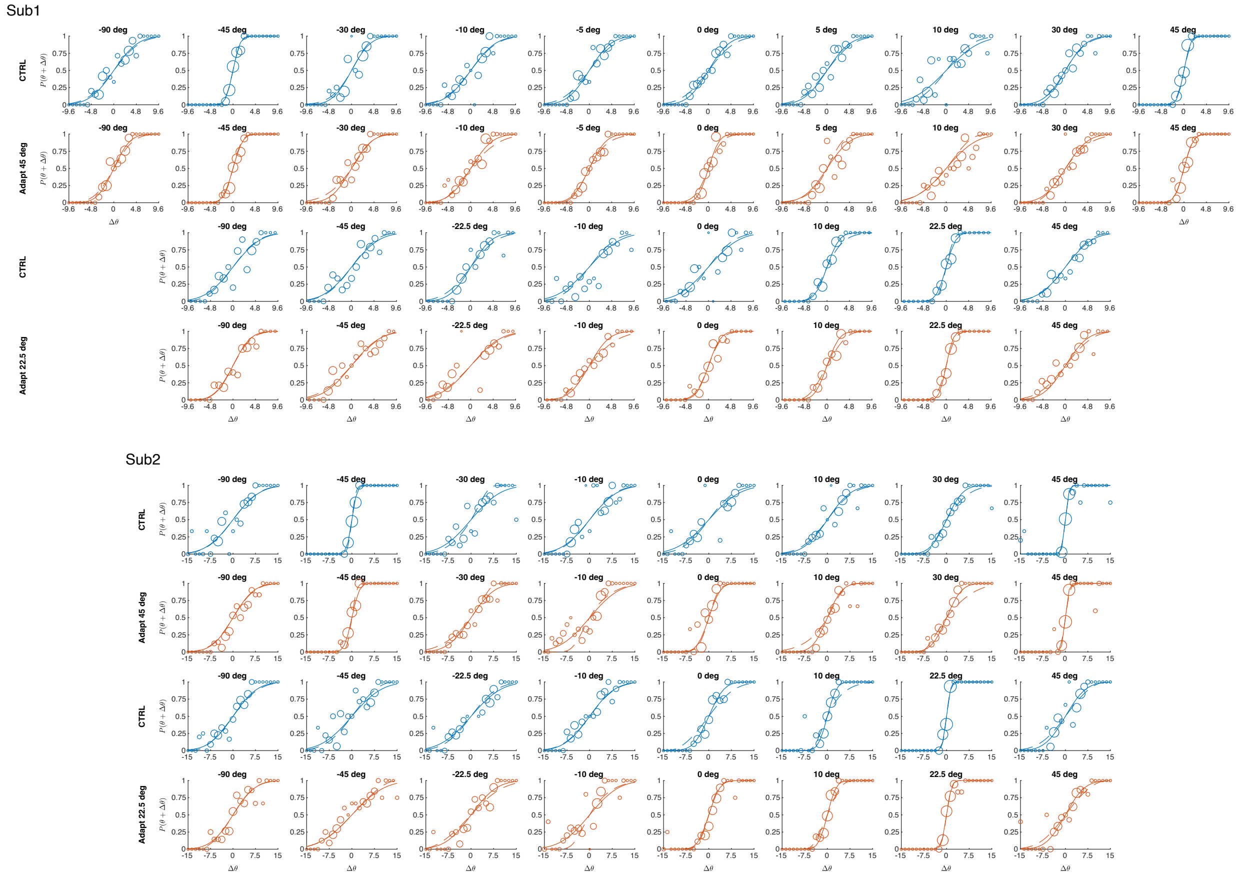

Supplementary Fig A: 2AFC response data of subjects 1 & 2 and psychometric curves fitted by a cumulative Gaussian distribution (solid line) or the reallocation 2-peak model (dashed line). Size of the data point represents the number of trials. Angle listed above each subplot indicates the difference between the test and the adaptor orientation.

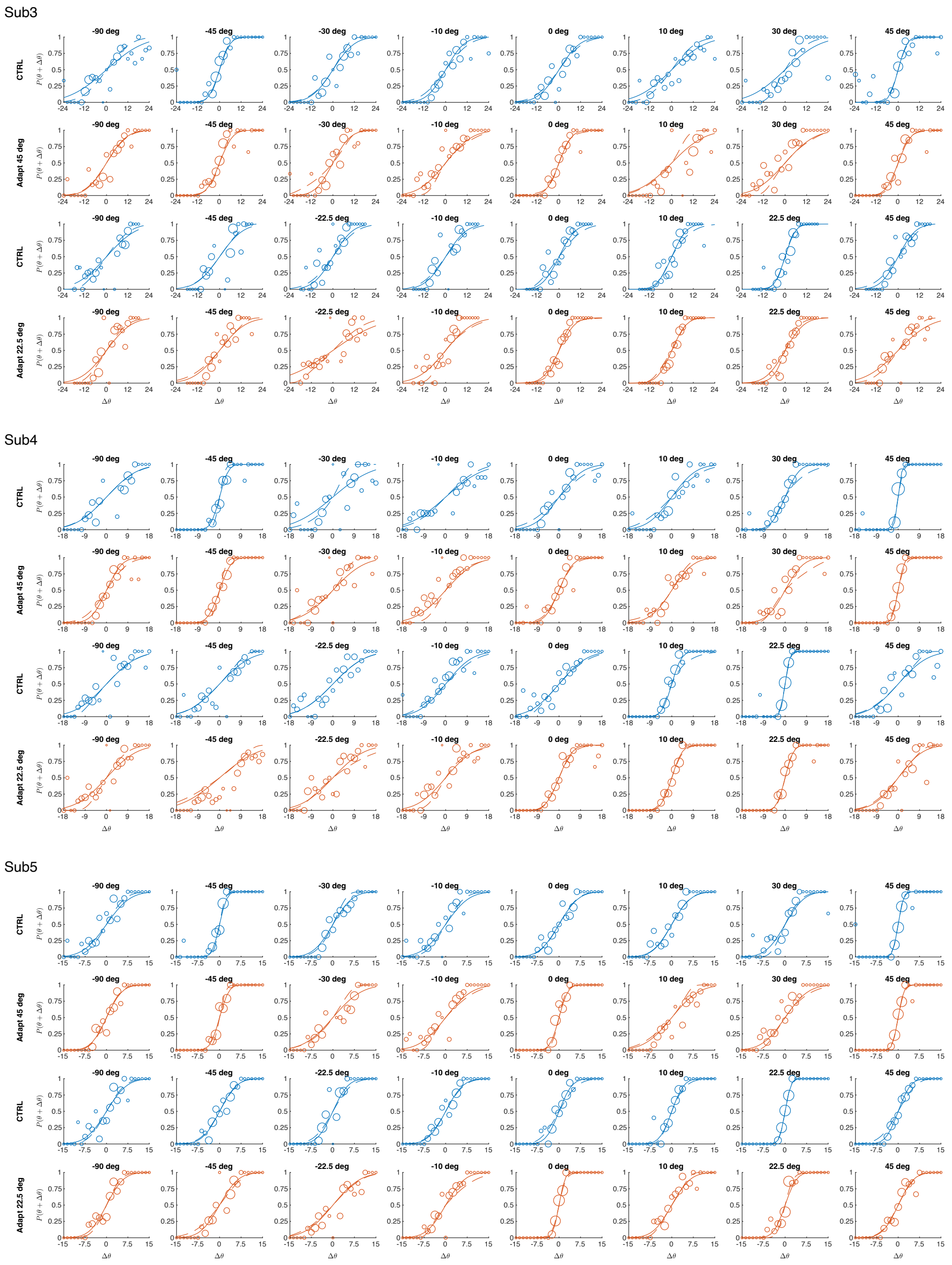

Supplementary Fig B: 2AFC response data of subjects 3, 4 & 5 and psychometric curves fitted by a cumulative Gaussian distribution (solid line) or the reallocation 2-peak model (dashed line). Size of the data point represents the number of trials. Angle listed above each subplot indicates the difference between the test and the adaptor orientation.

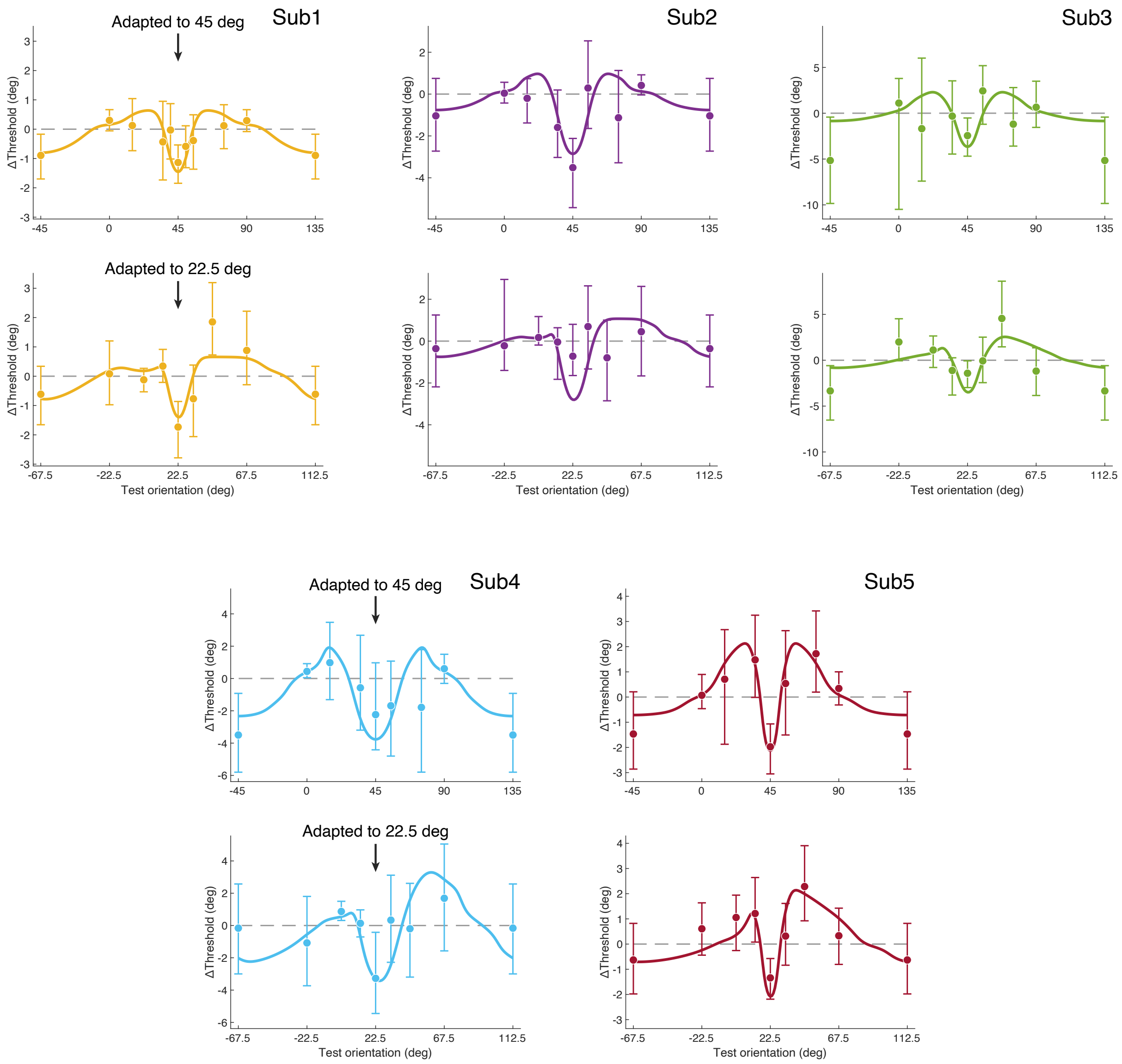

Supplementary Fig C: Difference in discrimination thresholds between control and oblique adaptor conditions for individual subjects. Solid lines correspond to the fit reallocation model. Error bars represent the 95% intervals computed over 1000 bootstrap samples of the data.

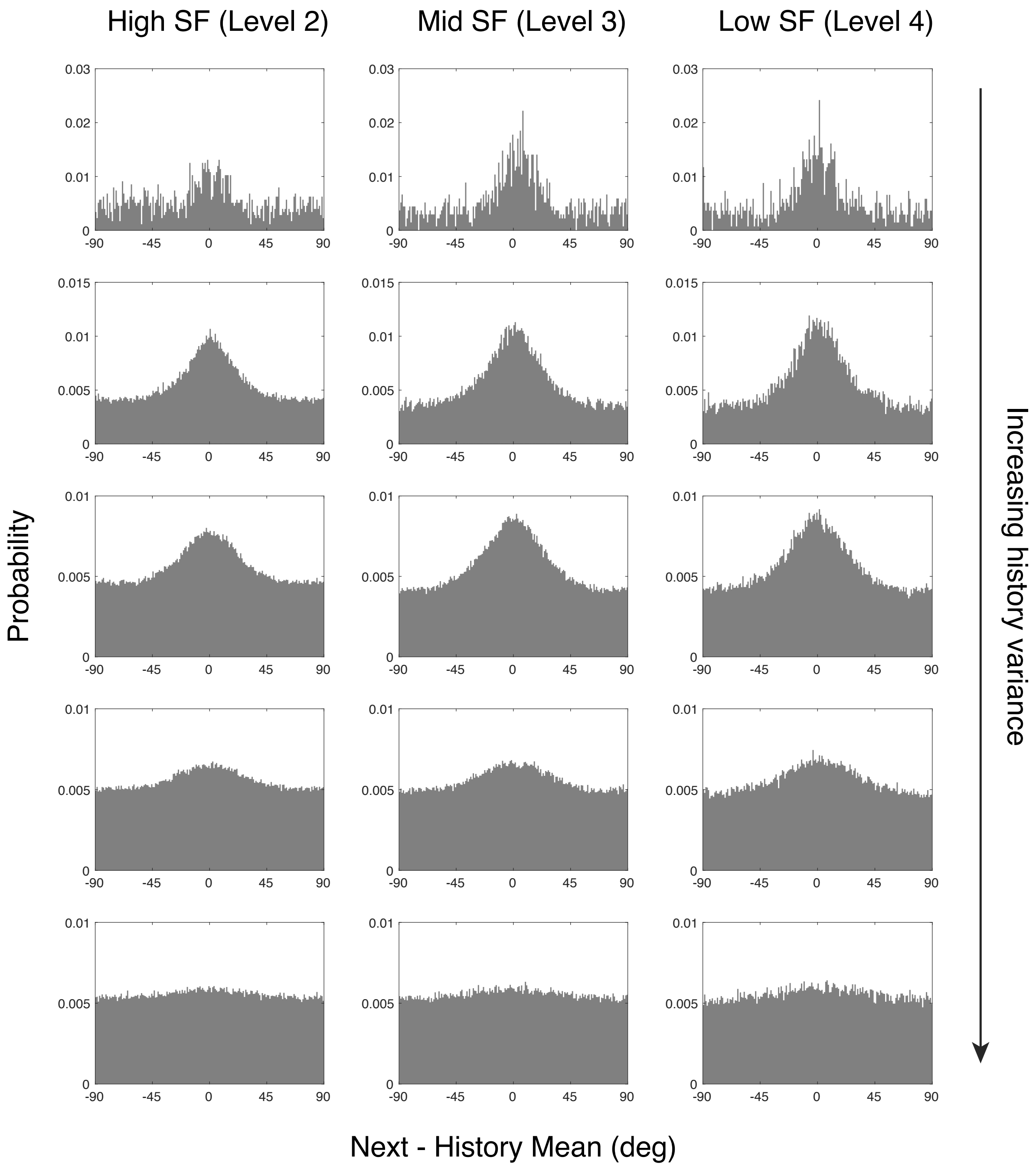

Supplementary Fig D: Distribution of orientation in the next frame relative to history mean (computed over 3s time-window) for different spatial frequencies and different history variance. The three spatial frequency levels were calculated from level 2 to 4 of the steerable pyramid and approximately correspond to 2.5, 1.25, and 0.63 cycles/deg, respectively. Variances are circular variances, ranging from 0 to 1 and binned into bins of size 0.2. For all spatial frequency levels, the distribution of orientation in the next frame is more concentrated around the history mean as the variance becomes smaller.
