# Supplementary material for: Adaptation optimizes sensory encoding for future stimuli
Source: PLoS Comput Biol. 2025 Jan 17;21(1):e1012746. doi: 10.1371/journal.pcbi.1012746 (PMC11771873; doi:10.1371/journal.pcbi.1012746)
Supplement: Attachment — Submitted filename: Adaptation_PlosCompBio_rebuttal_revA.pdf [file pcbi.1012746.s002.pdf]

Philadelphia, October 30, 2024

Dear Reviewers,

Thank you for your thoughtful comments and suggestions. Inspired by your feedback, we have thoroughly revised the manuscript, with the most substantial changes consisting of:

- a better description of the reallocation model, including an improved illustration (Fig.1) and a more explicit formulation of the connection between efficient coding (Fisher information), perceptual discriminability, and stimulus distribution (Eqs. 1 and 2).
- an improved presentation of the psychophysical data by showing discriminability (1/D) instead of thresholds D (Fig. 3 and Fig. 4), which allows a direct comparison to predicted encoding precision.
- a revised Discussion section.

We think these changes have significantly improved the paper in terms of clarity of presentation and the implications of our results. In addition, we have made small wording changes throughout the manuscript, and added also some references that were missing. Point-by-point responses to your specific comments are provided below.

Sincerely,

Jiang Mao, Constantin Rothkopf, and Alan A. Stocker

Reviewer 1

The paper argues that perceptual adaptation in humans is optimized to accurately represent expected, future stimuli. This work is a very nice mix of different approaches: theoretical results, human psychophysics, analysis of natural stimulus statistics, and machine learning models of perception. Overall, I find these results interesting and appreciate the variety of techniques and methods used by the authors.

Thank you.

My biggest concern is that it is not immediately clear how different parts of the manuscript relate to its title, i.e., the predictive nature of perceptual adaptation, and how they relate to each other.

I also think that the text is not clear enough and that the authors should significantly improve the presentation to facilitate the understanding of these results.

We appreciate your concern. We have substantially revised our manuscript and believe that our descriptions now clearly express the reallocation model and how it links to changes in encoding precision tailored for future stimuli in the context of long- and shortterm stimulus distributions.

Below I present some more specific concerns and suggestions:

1. The theory does not explicitly focus on the temporal dynamics of sensory stimuli. Please explain and clarify where the titular "future stimuli" are in the formalism. How do adaptation kernels and resource reallocation relate to prediction? In the current presentation, it is difficult to understand how the theory accounts for the dynamic nature of the stimuli. Where does the anticipatory/predictive component come from? I would welcome a better, more explicit explanation of these aspects.

The reallocation model assumes that encoding resources are reallocated according to an adaptation kernel, but does not a priori specify what that the form of the kernel is. We determine the shape of the kernel by fitting the model to our obtained psychophysical adaptation data. Then, with the statistical analysis of the natural retinal input stream, we show that the extracted kernel is in qualitative agreement with the statistical distributions of the orientation of future stimuli observed under similar temporal adaptation contexts as tested in the psychophysical experiments (i.e., after prolonged exposure to a low-variance (narrow-band) orientation history).

The reallocation model as presented is currently a descriptive model and not yet a full theory that makes precise quantitative predictions of how encoding changes given the specific stimulus histories. Also note that we do not make claims that adaptation "predicts" future stimuli. Rather, we demonstrate that adaptation induced changes in sensory encoding precision that are tailored for the stimulus parameter (orientation) in the very next visual input; thus the model accounts for the dynamic nature of the stimuli in a statistical sense (i.e., the distribution of the stimulus parameter value given the adaptation history).

2. The first part of the results (theory) is written in a very hermetic language that is not aimed at an interdisciplinary audience. For example, it is not at all clear what the difference between the "sensory space" and the "stimulus space" is. The authors refer a lot to the literature (including their previous work), but this cannot replace a good explanation of their theory. I suggest rewriting this section and giving more space to explaining the goal of the theory (e.g. in the current explanation, Fisher information suddenly changes to maximizing mutual information – line 128). I also suggest adding a diagram to Figure 1 explaining what stimuli are represented, what a "sensory space" is, etc.

Thank you for pointing this out. We have substantially revised the Model/Theory section to make it accessible and clear. We put special emphasis on explaining the concept of the sensory space.

We now also explicitly express the link between encoding precision (expressed in square-root of Fisher information) on one side, and psychophysically measured discrimination threshold and the stimulus distribution on the other, with Equations 1 and 2. Furthermore, we updated Figure 1 to better illustrate this relations in the context of the reallocation model and the adaptation states considered in the paper.

3. Perceptual experiments are well captured by the theory, but again: how do they relate to temporal prediction? It is not explained with the necessary clarity. The reader is left to guess.

The psychophysical experiments were performed to obtain a careful quantitative characterization of how adaptation changes encoding precision, i.e., the adaptation kernel. The link to the orientation distribution in the next frame after adaptation (future stimuli) is only established once we analyze the natural retinal video streams. Again, the hypothesis is not that adaptation predicts future stimuli but rather that it improves sensory encoding of the statistically more probable, next stimuli.

4. The section on natural scene statistics feels almost incomplete. Since a large part of it is devoted to describing the mechanics of computing orientations in natural video, it reads like a part of methods rather than results – the actual result is only two sentences (lines 225-230). The terminology in Figure 6 (e.g., "high history variance" vs. "low history variance") is very specific to the image analysis method rather than results. What is also missing is an explicit connection – either mathematical or even graphical – to the theory presented in the first part. How do these statistics relate to theory, psychophysics, and prediction? There is a statement about adaptation kernels, but it is very verbal, short and unclear. I believe this section should be significantly improved.

We have substantially revised and extended this section. Specifically, we clearly specify in the first paragraph the purpose of analyzing the natural retinal videos: "We extracted the orientation distribution in the next image frame after the human observers naturally experienced prolonged exposure to retinal input with a relatively static orientation content. The goal was to test whether these distributions match the changes in encoding precision (adaptation kernels) we extracted from the psychophysical adaptation data, and thus support the hypothesis that adaptation optimizes sensory encoding for statistically more likely, future stimuli." We think, in combination with revised Fig.1, the link between stimulus statistics and adaptation kernel is now much clearer established.

5. The same criticism applies to the description of PredNet. I appreciate the Fisher information analysis of the network. However, it is presented almost as an independent fact. In my opinion, it should be clearly explained how the goal of PredNet (reconstructing the next frame of a movie) is related to the theory presented (Fisher information maximization) and the perceptual results. I don't think this is done sufficiently well at the moment.

We appreciate your suggestions. We think our focus on Fisher information as a unifying metric of

encoding precision is a major strength of the paper. Starting from the Introduction, we use Fisher information to quantitatively tie together efficient coding, psychophysical discriminability, natural image statistics, and encoding precision in an recurrent neural network. With the revision, we have further improved and clarified this connection (see Eqs. 1 and 2, for example). We hope that the new format of presenting the psychophysical data (Fig. 3 and 4), showing discriminability instead of discrimination threshold, also helps the reader to better connect theory and data.

Reviewer 2

The authors argue that the goal of sensory adaptation (to orientation) is to allocate information processing resources to likely upcoming visual input. They use psychophysical data and visual scene statistics in the presence of eye movements to support their argument. In addition , they demonstrate that a recurrent artificial neural network designed to predict the next input also shows a similar pattern of adaptation.

The strength of the study is that concepts that have been linked informally in the past, are linked explicitly, with a solid computational framework, and strong supporting experimental data.

Thank you.

My comments are mainly requests for clarification and discussion:

The measured thresholds and fits support the main hypothesis. However, thresholds were not always collected at all relevant test orientations (and this seems to have varied across subjects and even adapters -see “missing” thresholds at some of the troughs of the fitted curves). Please explain why. Obviously this is not ideal as it implies that the trough is driven solely by model assumptions of symmetry). This is further complicated by the observation that most subjects show asymmetries in the thresholds that are not well captured by the hypothesis (e.g., higher thresholds on one “side” of the adapter than the other. I’d recommend doing a (model-free) analysis of the thresholds at the single subject level to document (a)symmetry and potentially identify other aspects of the data that are statistically reliably but not capture by the normative model. This does not distract from the overall value of the normative model, but provides nuance to the claim that adaptation is only about optimizing for the future input.

We appreciate and understand the reviewer’s concern. Ideally we would have liked to measure discriminability at as many orientations as possible to get an as accurate as possible estimate of the sensory encoding space. Practically, however, adaptation experiments are quite time consuming and difficult to perform correctly, and thus we had to limit the number of test orientations. We measured at symmetrically spaced, relatively densely sampled test orientations relative to each of the two oblique adaptors (see Methods for details). All subjects were measured at the same test orientations, with subject 1 also tested for two additional test orientations for the 45 deg oblique

adaptor condition.

Besides not having a data point at 90 deg in the 22.5 deg oblique adaptor condition, we sample all locations where the model makes strong predictions (around the adaptor and orthogonal to it). Furthermore, the missing data point is at a cardinal orientation and far away from the adaptor orientation, and thus likely not affected by adaptation. Note that discriminability in the control adaptor condition is measured at more stimulus locations because we measured it at the same test locations that we measure both the 45 and 22.5 deg oblique adaptor.

Regarding asymmetries, we do not quite understand the reviewer's concern. The asymmetries (different discriminability on both side of the adaptor) in discriminability after adaptation are well predicted by the model (see Fig.1 - model fit is mostly within the errorbars), because the kernel is only symmetric in the sensory space and the resulting effect in stimulus space depends on the adaptor orientation. For reasons of simplicity, we intentionally did not model any potential asymmetry between encoding precision for the two cardinal orientations in the control condition; some subjects show a slightly higher precision for vertical orientations (0 deg), some do not. We explain this in the revised description of the results.

The description of the histories is not quite clear. A sentence like L279-L281 seems tautological in isolation. Pleas explain how time (or space?) windows were selected to generate the low/high variance conditions. Why are the distributions for different spatial frequencies relevant for the case made in this manuscript? A sentence or two on the nature of the different levels of the steerable pyramid would also help the reader.

The reviewer raises a good point. Under natural free-viewing conditions, subjects are only very rarely experiencing the same, 60 seconds long adaptation condition as in our psychophysical experiment. As a result, computing the conditional distribution of the orientation in the next frame given the stimulus history over such a long time-window for a very narrow adaptor distribution is not possible. Thus, we resorted to a compromise of having a still relatively long time-window that provided sufficient data to compute a good estimate of the distribution. We explain this in the revised manuscript (line 236ff).

Statistical analyses of orientation distributions in natural images depend somewhat on the filter design, and there in particular on the resolution (i.e., the spatial frequency; see, e.g., Supplementary Fig.4 in Girshick et al. (2011)*. Given that our work is not addressing neural adaptation in a particular visual area, the relevant spatial scale is unspecified. Therefore, we computed the statistics over all scales to demonstrate that our conclusions are not dependent on spatial frequency.*

We added a short description of the steerable pyramid in the Method section.

L305. Is the contrast of the control adapter really identical for a v1 receptive field? To me this seems unlikely as the combination of orientations tends to cancel out some of the peaks and

troughs. Please quantify.

As mentioned above, our analysis is not specific to any particular neural processing stage. As such, the stimulus design was not specific to address adaptation of, e.g., V1 neurons. Adaptors were matched in terms of their stimulus properties (spatial frequency spectra).

Figure8 – it may be helpful to draw a thin blue line (representing the control condition) in the adaptation panels of b (it took me a while to realize that the orthogonal directions were indeed reduced relative to control).

Thank you for the helpful suggestion. We have added the line.

Figure5 - 100 bootstrap samples - that seems an unusually small number for a confidence interval. What motivated the choice to use four "adaptation" frames in PredNet? Is there some way to relate this to time? Does the effect change with the number of frames?

We have now doubled the number of bootstrap samples (200). Note that the resulting confidence intervals essentially did not change.

*Ideally, we would have tested PredNet with the same adaptor duration as in our psychophysical experiments. However, that was computationally too costly (60s * 24 frames = 1440 frames) given our sampling based analysis (200 stimulus samples). We tested 10 adaptor frames and found that the results were quantitatively very similar to the current results based on 4 frames. Therefore, we consider 4 frames sufficient, in particular given that our comparison with the psychophysical data and the retinal input statistics had to remain somewhat qualitative. We revised the Method section accordingly.*

Reviewer 3

This work examines the phenomenon of adaptation to visual sensory stimuli. The authors measure behavioral adaptation in psychophysics experiments involving subtle discrimination of oriented bars. Participants show sharper psychometric curves around the target (adapted) orientation but also around the orthogonal orientation. The authors finally use a predictive coding model to capture the behavioral effects. There also an interesting measurement and discussion of natural image statistics and dynamic changes through head mounted displays that include eye tracking. This part is quite interesting but not well integrated with the rest of the paper (not even mentioned in the abstract, except in a highly indirect manner). Overall, the results are interesting and provide insights about the mechanisms underlying adaptation in sensory processing.

Thank you.

1. If I understand Fig. 1 correctly, the sensory enhancement (middle row) at the adapted orien-

tation is narrow whereas the corresponding enhancement at the orthogonal orientation is rather broad (according to the reallocation model). a. The caption to Fig. 1 seems to suggest that Fisher information (J(theta)) is shown, but the y-axis in the figures says Fisher sqrt(J). In any case, there is no real quantitative y-axis, so the figures could be reflecting J or sqrt(J) or any other function. b. It would be nice to explain why is this the case c. Is it possible to test this prediction experimentally as well? d. The y-axis in Fig. 1 does not have any numbers so I am not sure how seriously one should take the idea beyond the conceptual level. In addition to the broadening, the peak seems to be larger at the target orientation compared to the orthogonal one. To the extent that the magnitude of this peak is relevant, one may deduce that there would be more modulation at the target orientation than at the orthogonal one. But this prediction does not seem to be supported by the results in Fig. 2.

We appreciate the comments/concerns regarding the model explanation and illustration. In the revision, we substantially revised both to resolve all these ambiguities. The fact that the illustrations show a broader coding enhancement at orthogonal orientations of the adaptor is not because that is hard-wired into the model, but because it resembles the resulting fit of the model to the data. The actual (fit) adaptation kernels for each individual subject are shown in Figure 5.

a) We now consistently plot encoding precision in units of square-root of Fisher information. This allows for a direct comparison to the stimulus distribution according to efficient coding (see Eq. 2). Please note, the graphs in Figure 1 are illustrations and thus we omitted numerical units.

b) We have carefully revised the Model/Theory section to explain why the model makes those predictions.

c) We test these predictions with our psychophysical adaptation experiments shown in the next section.

d) As mentioned above, Figure 1 represents a conceptual illustration albeit based on an actual model instance. The specific form of the resource reallocation (i.e., the square-root of Fisher information) is determined by the fit to the data (Figure 5). The magnitude of the peak is insofar relevant as the total encoding resource is assumed to be constant. As our results convincingly show for all subjects, the change in coding precision is by far largest for orientations that equal the adaptor orientation.

We hope that the revised section now clarifies these questions.

2. The test in Fig. 2 involves some parameter choices. While I am perfectly ok with those choices, I wonder how the authors decided them. Did they try different parameters and converged on a set of parameters that work. Would the results of adaptation be stronger with longer adapting times? The authors discuss eye tracking in the context of other experiments but do not seem to be mention whether subjects maintained fixation during the entire 1 min of adaptor time + 5s top-up

adaptor.

Thank you for raising this point. We picked an adaptation duration of 1 min because i) the magnitude of adaptation induced changes are well saturated and do not substantially increase for longer durations and ii) it represent a common choice in previous studies and thus allows comparisons to existing data. We have updated the Method section to more explicitly express the rationale of our choice.

Regarding eye-tracking: We did not control for fixation. However, subjects were instructed to maintain fixation throughout the initial adaptation phase and during each trial (which includes the top-up phase). This is described in the Methods section.

3. I wonder whether models that incorporate adaptation would reproduce the experimental results in Fig. 2/3. For example, Vinken et al 2020 (Incorporating intrinsic suppression in deep neural networks captures dynamics of adaptation in neurophysiology and perception) developed a model that incorporated adaptation into a visual recognition neural network. I guess that this type of model would reproduce the effects at target orientation but I am not sure about the orthogonal one. Similar questions apply to other adaptation models.

This is a good question. However, the focus of our study was not to test to what degree artificial neural networks with adapting neurons show encoding changes similar to those we measured in human subjects (our guess is 'yes'). Rather, our rationale for analyzing PredNet was specifically to test whether i) a network optimized to predict the future would show such encoding enhancement, and ii) would show them without having adaptive neurons. We do agree that analyzing the computational properties of ANNs with adaptive neurons is an interesting future direction.

4. The authors have not made data or code available. They indicate that they will upon acceptance. In my opinion, thisi not ideal practice and code/data should be submitted for review as well.

Code and data are now available for the reviewers at this private Google Drive folder. Please, do not share. As originally stated, we will publicly share both at an open access GitHub repository upon acceptance of the paper.

References

A. R. Girshick, M. S. Landy, and E. P. Simoncelli. Cardinal rules: visual orientation perception reflects knowledge of environmental statistics. *Nature neuroscience*, 14(7):926–932, 2011.
